# Scoring Function Based on Weighted Residue Network

**DOI:** 10.3390/ijms12128773

**Published:** 2011-12-02

**Authors:** Xiong Jiao, Shan Chang

**Affiliations:** 1 Institute of Applied Mechanics and Biomedical Engineering, Taiyuan University of Technology, Taiyuan 030024, China; 2 College of Informatics, South China Agricultural University, Guangzhou 510642, China; E-Mail: schang@scau.edu.cn

**Keywords:** residue network, weighted parameter, protein-protein docking, scoring function

## Abstract

Molecular docking is an important method for the research of protein-protein interaction and recognition. A protein can be considered as a network when the residues are treated as its nodes. With the contact energy between residues as link weight, a weighted residue network is constructed in this paper. Two weighted parameters (strength and weighted average nearest neighbors’ degree) are introduced into this model at the same time. The stability of a protein is characterized by its strength. The global topological properties of the protein-protein complex are reflected by the weighted average nearest neighbors’ degree. Based on this weighted network model and these two parameters, a new docking scoring function is proposed in this paper. The scoring and ranking for 42 systems’ bound and unbounded docking results are performed with this new scoring function. Comparing the results obtained from this new scoring function with that from the pair potentials scoring function, we found that this new scoring function has a similar performance to the pair potentials on some items, and this new scoring function can get a better success rate. The calculation of this new scoring function is easy, and the result of its scoring and ranking is acceptable. This work can help us better understand the mechanisms of protein-protein interactions and recognition.

## 1. Introduction

Protein-protein docking is an important method for the protein-protein interaction and molecular recognition [1–6]. The aim of the protein-protein docking is to forecast the structure of the complex based on the structure of the two monomers. After an efficient search for the conformation space, we need a rational and sensitive scoring function to evaluate the docking results [7,8]. With an efficient docking scoring function, we can distinguish a correct docking binding pattern from the incorrect ones. Finally, some native-like structures can be picked out from the decoy set [3]. In order to get a good scoring and ranking result, the docking scoring function needs to take some important elements into account, for instance, the geometry complementarity [9] and potential energy functions [5,10–12]. The potential energy contains the influence of different factors, such as the residue pairing preference, static electricity, hydrogen bond and hydrophobic interaction. Unfortunately, some calculations of these factors are related with the atoms’ location. So, the calculation of these potential energy functions will be time-consuming, and the computational accuracy of these functions will also be affected by the accuracy of the structure data. Based on the interaction between residues, some new docking scoring functions have been developed, and these functions can be calculated quickly [5,10,11,13–15]. For the binding between proteins, different binding modes have different interface characteristics, such as the composition of different types of amino acid, the hydrophobicity and electrostatic potential. The research of the docking scoring function has made some progress. However, due to the various binding modes of different complex systems, there is still no suitable scoring function for the protein docking of all types’ molecular systems.

From the view point of complex network [16–18], we can treat a protein molecule as a complex network [19–23]. With the aid of the network model, some research has produced a few meaningful results. Most of these studies are related to the protein folding or the relation between structure and function. Such works include: the identification of the key residues for a protein through the residue network parameter–betweenness [19]; through the measuring of the topology for the protein contact network, the topological property of the protein conformation is shown to have an influence on the kinetic ability when the protein folding [24]; the average shortest path length is found to have a high correlation with the residue fluctuations [25]; the active site residues can be identified through the network parameter–closeness [21].

In the residue network model, we simplify each residue to a single point, and this point is used to be the node of the network. Based on the distances between these points, the links between them can be set. If the distance between two nodes is less than a cut-off value, then there will be a link between these two nodes [26]. Consulting the various interaction situations between different residues, we can assign appropriate weights to the network links. The weight can be the number of atom contacts between nodes [26], or it can be the probability of the contact between amino acids [19]. In this paper, we use the contact energies as the link weight [27]. The contact energies can reflect the various interactions between residues.

In our previous works, we have used the residue network to do the protein-protein docking scoring [13,14]. In those works, we divided the residue network into some sub-network according to the type of residue (hydrophilic, hydrophobic and neutral), and proposed some scoring terms based on the unweighted network parameters. We also researched the network rewiring on the binding interface when two monomers interacted.

When we consider the contact energy between residues, we can get some interesting results [28]. This weighted network model does not need to make a distinction between the types of residue. With this weighted residue network model, an exploratory work on the docking scoring function [29,30] is presented in this paper, and a new scoring function is proposed. In order to realize a quick calculation for the scoring rank, the types of the docking system are not distinguished in this scoring function.

## 2. Material and the Research System

From the data set of protein-protein docking benchmark 2.0 [31], we select 42 dimer complexes to do the docking calculation.

In the docking benchmark 2.0, there are four types of test cases: enzyme-inhibitor, antibody-antigen, other and difficult test cases. For the antibody-antigen complexes, there are some complementarity-determining regions, and the binding modes in the antibody-antigen complexes are relatively fixed. So we did not include the antibody-antigen complexes in our test.

In the remainder of test cases, only the single-chain monomer structures for ligand and receptor were selected to do the docking and the residue network analysis. These 42 complexes can be classified into two groups. The ‘Enzyme-Inhibitor’ group contains 18 complex structures. The ‘others’ group contains 24 structures. The size of these protein complexes is from 185 to 1100. With the RosettaDock 1.0 program [32], we do the bound and unbound docking calculations for all 42 systems. In each case, we generated 1000 structures for farther calculation.

## 3. Theory and Method

In the residue network, the geometrical center of each amino acid’s side chain is chosen to act as network node. The link between a pair of nodes is determined by the distance between these two nodes. If the distance between residues *i* and *j*, marked with *r_ij_*, is less than the cut-off (*r_c_* ) value of 6.5 Å, then there will be a link between these two residues. Thereby, the unweighted residue network can be given and its adjacency matrix element can be expressed as follows:

(1) $$a_{ij}=\{\begin{array}{cccc} 1 & i\neq j & and & r_{ij}<r_{c}\\ 0 & i=j & or & r_{ij}\geq r_{c} \end{array}$$

Based on the contact energies between residues, the weighted network can be constructed, and its adjacency matrix element can be expressed as:

(2) $$a_{ij}^{w}=\{\begin{array}{cc} a_{ij}w_{ij} & j\neq i\pm 1\\ 2.55 & j=i\pm 1 \end{array}$$

where *w_ij_* is the link weight. According to the magnitude of the contact energy between residues *i* and *j* suggested by Miyazawa *et al*. [27], appropriate weights are assigned to the network links. These link weights are related with the types of these two amino acids. To avoid a negative weight value, the absolute value of the contact energy is used as the weight.

For the contact energies between residues used in this work, all the values of the contact energies are less than zero. In other words, all the energies in this set of potential are negative value. So, the use of the absolute value of the contact energy is reasonable, and we can do the addition and subtraction between these absolute values.

For the covalent bond between residues *i* and *i ±* 1, the link weight is assumed to be 2.55, which is the absolute value of the average collapse energy [27].

Additionally, a new network parameter–strength (marked with S) is introduced into the weighted residue network. The definition of strength of node *i* can be written as [33]:

(3) $$S_{i}=\sum_{j=1}^{N}a_{ij}^{w}$$

where *N* is the number of network nodes. *a_ij_^w^* is a matrix element of the weighted adjacency matrix. Strength of node *i* (*S_i_*) represents the sum of the link weights, which belongs to the link with node *i* as an endpoint. Furthermore, the strength of the whole network ( *S* ) can be defined as the expression in Equation 4. This parameter represents the sum of all link weights of the network model.

(4) $$S=\frac{1}{2}\sum_{i=1}^{N}S_{i}$$

For the contact energy between residues suggested by Miyazawa *et al*., the stronger the non-covalent interaction between two residues, the more the link between residues contributes to the stability of the whole protein. The link, corresponding to a stronger interaction between residues, will get a bigger weight in the residue network model. For the strength of the whole network, a more stable protein will obtain a bigger strength than an unstable one. So, we can use this parameter to evaluate the stability of the protein.

In the unweighted network model, the average nearest neighbors degree *k_nn_*_,_*_i_* is defined as [33]:

(5) $$k_{nn,i}=\frac{1}{k_{i}}\sum_{j=1}^{N}a_{ij}k_{j}$$

where *a_ij_* is an adjacency matrix element of the network, *k_i_* is the degree of node *i*. When link weight is introduced into the residue network model, the weighted average nearest neighbors degree *k_nn_*_,_*_i_^w^* can be defined as [33,34]:

(6) $$k_{nn,i}^{w}=\frac{1}{S_{i}}\sum_{j=1}^{N}a_{ij}w_{ij}k_{j}$$

where the meaning of *S_i_*, *a_ij_*,*w_ij_*,*k_i_* is the same as the definition mentioned above. The superscript *w* means that it is for the weighted network model. In this weighted model, we use a standardized coefficient to calculate the nearest neighbors degree. This standardized coefficient is based on the weight of the link. So, we can define the weighted average nearest neighbors degree 〈*k_nn_^w^*〉 for the whole weighted network:

(7) $$\langle k_{nn}^{w}\rangle =\frac{1}{\text{N}}\sum_{i=1}^{N}k_{nn,i}^{w}$$

where *N* is the number of network nodes, and *k_nn_*_,_*_i_^w^* is the nearest neighbors degree of node *i*.

The nearest neighbor degree of a given node measures the effective attraction of this node to connect with its environment. For nodes with a high degree or low degree in the environment of a specific node, the weight of the interactions is taken as a referential meaning. The weighted average nearest neighbors degree of the whole network measures the weighted assortative or disassortative properties of the whole weighted network, also with the weights of actual interactions among nodes as a referential standard. This parameter can be used to evaluate the connection mode between different nodes with various degrees.

When we get the docking results, we superimpose the receptors of the decoy onto the native structure, so the RMSD of the ligand (L_RMSD) over its backbone atoms (*N*,*C*,*C_α_*,*O*) can be calculated. The near-native structure, or the hit structure, is defined as the one with L_RMSD ≤ 4.0 Å for the docking structures.

**Figure 1:**
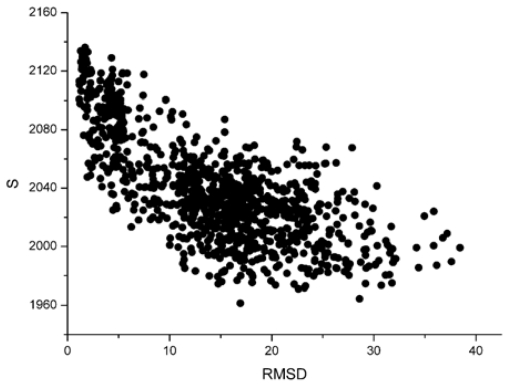
The relationship between the whole strength of the network (S) and the L_RMSD (The 1udi was taken as an illustration).

**Figure 2:**
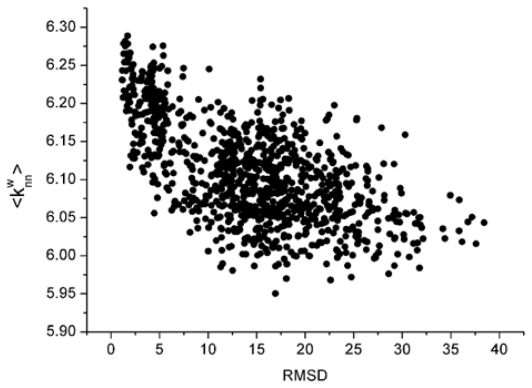
The relationship between the weighted average nearest neighbors’ degree and the L_RMSD (The 1udi was taken as an illustration).

For these decoy structures, we analyze two parameters of the weighted residue network: the strength ( *S* ) and the weighted average nearest neighbors degree of the whole network (〈*k_nn_^w^*〉). And then we calculate the correlation between the *S* and L_RMSD and that between the 〈*k_nn_^w^*〉 and L_RMSD. With the unbound decoys of 1udi as an illustration, the results are shown in Figure 1 and Figure 2.

From these two figures, we can find that *S* and 〈*k_nn_^w^*〉 all have a negative correlation with the L_RMSD for the 1udi system. For the calculation results of other different protein systems, it is very similar with that of 1udi. So, each of these two parameters has a discrimination power of docking decoys, and we can use a combination of them to act as a scoring function.

For the parameter *S*, the dissimilarity between different systems is notable. The parameter ‘strength’ has relation with the size of the system. However, the sizes of various systems are distinctly different. So, these strengths are obviously dissimilar. But for the weighted average nearest neighbors degree 〈*k_nn_^w^*〉, there is no a remarkable difference. The value is about 6 for all the 42 systems.

So, if we use a linear combination of these two parameters, the relative size of these two items will change dramatically with the system size. For a bigger system, the whole strength of the network will have a bigger contribution than that of 〈*k_nn_^w^*〉. For a small system, the situation is just the reverse. In order to balance the relative size of *S* and 〈*k_nn_^w^*〉, we need to adjust the linear combination coefficient with the sizes of different systems. This adjustment needs a heavy calculation, and it is time-consuming. Then, as an alternative choice, we take a nonlinear combination–the product of these two parameters–as the scoring function to do the scoring and ranking for the docking results. In order to compare with other scoring functions, in which a better docking decoy will get a lower score and can be ranked ahead in the scoring rank sequence, a negative value of this product is adopted. With this scoring function, the docking decoy with a small L_RMSD, which is regarded as a better docking structure, will be ranked ahead. We label this scoring function as *S_n_*:

(8) $$S_{n}=-S\times \langle k_{nn}^{w}\rangle$$

## 4. Result and Discussion

For all 42 systems, we do a bound and unbound docking calculation in this paper. In order to assess the quality of this scoring function, we use some indicators to evaluate its discriminative ability for the docking decoy, such as: the correlation coefficients between the scoring values and L_RMSD; L_RMSD of the first rank; rank of the first hit and number of hits in top 10 scores. All these four indicators are commonly used in the evaluation process of other docking scoring functions. We select the pair potentials (RP) scoring function [35] to do the comparison with this new scoring function. As pair potentials, RP is based on the residue pairing preference in the interface, so it is reasonable to make a comparison between RP and this new scoring function on the residue level. On the other hand, RP is widely used in the relative works, and is adopted by the FTdock program. For all 42 systems, the four items mentioned above are calculated. The comparison of these indicators is carried out between the scoring results of the Sn and those of the RP.

**Figure 3:**
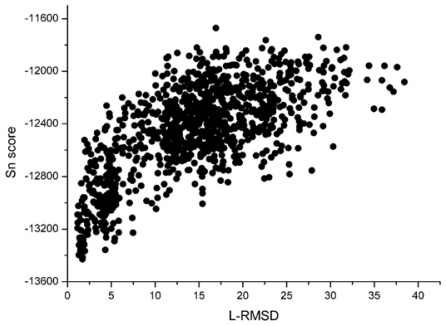
The correlation between the L_RMSD and the Sn scoring function (The 1udi was taken as an illustration).

**Figure 4:**
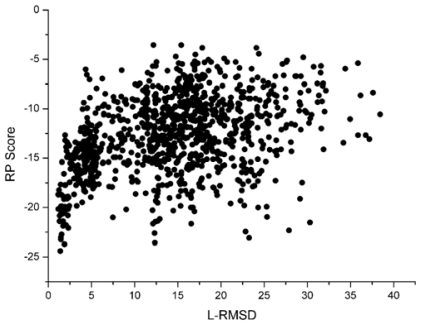
The correlation between the L_RMSD and the RP scoring function (The 1udi was taken as an illustration).

With the 1udi system as an illustration, Figure 3 shows the scoring rank result of unbound decoys with the Sn scoring function. The corresponding result of RP scoring is shown in Figure 4. From Figure 3, we can see that the decoy with low L_RMSD will get a low scoring value. So, it will be rank ahead in the scoring sequence from Sn. Apart from for the scoring results of RP, as shown in Figure 4, some decoys with high L_RMSD will also get a low scoring value.

For the correlation coefficients between the scoring values and L_RMSD, it reflects the scoring results from the point view of whole, and a higher correlation coefficient value is more accepted. Through the comparison between Figure 3 and 4, we can find that the correlation got from Sn scoring function is higher than that of the RP score. For the 1udi system, Sn has a better performance than RP on the indicator–correlation.

**Figure 5:**
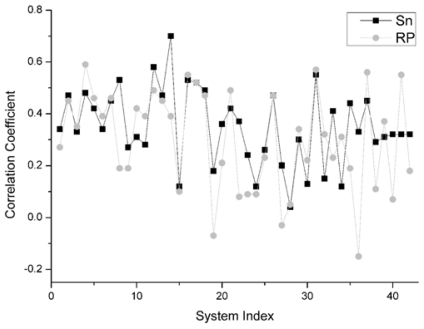
For the unbound dock results, the comparison of the correlation between the score value and the L_RMSD for Sn and RP score.

For the unbound decoys of all 42 systems, the comparison results on this indicator between Sn and RP is shown in Figure 5. From this figure, for all 42 systems, we can find that there are 22 systems on which Sn gets a higher correlation coefficient value than the RP scoring function. Moreover, there are two systems and the Sn gets the same results as the RP scoring function. So, we can conclude that Sn has a similar performance to RP on the indicator–correlation coefficient.

For the number of hits in top 10 scores, it measures the discriminatory power of the scoring function to pick hits out in their top 10 scores. This indicator is a most indicative one, so this parameter is commonly used to measure the ‘success rate’ in docking. The more hits that are picked out, the more preferable the scoring function can be considered.

**Figure 6:**
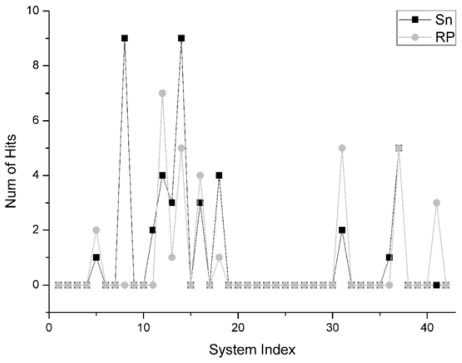
For the unbound dock results, the comparison of the numbers of the hit structures of the Sn and RP score function.

There are 30 systems on which the Sn and the RP do not pick the hit out in their top 10 scores. In the remaining 12 systems, there are six systems on which the Sn gets more hits in their top 10 scores than that of RP, and there is one system that the Sn gets the same account of hits as RP. All the comparisons regarding the number of hits are shown in Figure 6. Especially for the system 1e6e and 1udi in the Enzyme Inhibitor group (as the two highest squares in Figure 6), the Sn gets nine hits in the top 10 scores. However, the results of RP are 0 and 5 respectively. So, on the whole, it can be concluded that the Sn has a similar performance with the RP on the number of hits in top 10 scores. However, Sn has a better discriminatory power than RP on some specific systems.

**Figure 7:**
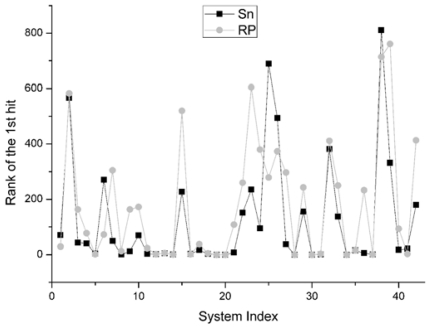
For the unbound dock results, the comparison of the rank of the first hit structure of the Sn and RP score function.

For the rank of the first hit, it reports the rank position of the best decoy in the scoring sequence. If the rank value is 1, *i.e.*, the best decoy has been picked out firstly by the scoring function, it will be thought as the best situation. The comparison result between Sn and RP on 42 systems is shown in Figure 7. We find that the results of Sn are superior to those of the RP scoring function. In all the 42 systems, there are 26 systems for which the Sn gets better results, or ahead rank positions for hits, than RP scoring function. In 8 systems, these two scoring functions get the same results. On this indicator, the Sn also has a better performance than the RP.

**Figure 8:**
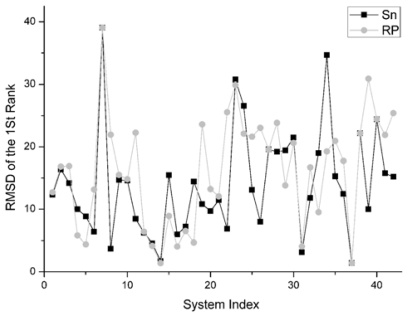
For the unbound dock results, the comparison of the RMSD of the first rank structures obtained from the Sn and the RP score function.

The RMSD of 1st rank reflects the quality of the first decoy in the rank sequence. The smaller the RMSD of the first ranked decoy, the better the scoring function performed. The comparison result on this indicator between Sn and RP on 42 systems is shown in Figure 8.

For the first rank structures, if its RMSD is larger than 10 Angstroms, this first rank structure should be thought of as wrong. Consequently there is no sense in comparing these structures.

There are 19 complexes with a low L_RMSD (less than 10 Angstroms) in Figure 8. For these 19 complexes, the Sn function gets smaller RMSD than the RP scoring in nine systems. The Sn function gets higher RMSD than the RP scoring in nine systems. In one system, these two scoring function get the same RMSD value for the first rank. So we can conclude that the Sn has a similar performance to the RP on the RMSD of the first rank.

From a global perspective, we carried out a performance evaluation of the Sn scoring function. As generally used in the related work, the success rate of a scoring function is used to measure its average ability to rank a near-native structure within some number of predictions (NP).

If we can find at least one near-native structure in the top NP decoys from the ranked queue, this case is defined as a successful case under NP. Then we can calculate the percentage of successful test cases in the data set. When the NP changed, we were able to get the success rate curve.

**Figure 9:**
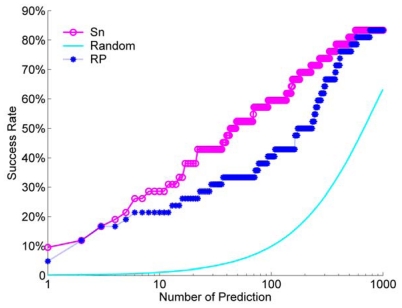
For the unbound dock results, the comparison of the success rate between the Sn scoring function, RP scoring function and a random rank.

We calculated the success rate for the Sn scoring function and the RP scoring function. For a comparison, we also did a random ranking for 100 times, and then calculated its average success rate. For the unbound decoys, the success rate of Sn, RP and a random scoring are shown in Figure 9.

From Figure 9, we can see that the Sn has a better performance than the RP at most NP. When we select NP as 10, the success rate is about 30%. This means that when we select the top 10 decoys from the ranking queue, we can find at least one hit with a probability of about 30%. Only at the beginning of the success rate curve, or when we select a few decoys (NP from one to three), the difference between Sn and RP is small. Sn and RP all have a much better performance than a random rank.

From these comparison results, as a whole, we can conclude that the Sn scoring function has a similar discriminative ability with the RP scoring function for unbounded docking decoy.

For the bound docking results, we also did the comparisons from these four point views. For the correlation coefficients between the scoring values and L_RMSD, we find that there are 22 systems on which Sn has a better performance than the RP scoring function. For the number of hits in top 10 scores, there are 18 systems on which Sn picks more hits out in their top 10 scores, and there are 15 systems that these two scoring function get the same amount of hits in the top 10. Sn has a powerful discrimination to pick more hits out in their top 10 scores than the RP. For the rank of the first hit, we find that there are 19 systems in which the first hit is ranked ahead by Sn than the RP score. In addition, there are 13 systems that the rank position of the first hit is same in the Sn as that in the RP. For the RMSD of 1st rank, there are 29 complexes with a small RMSD (less than 10 Angstroms). For these 29 systems, there are 16 systems in which the Sn function gets smaller RMSD than the RP scoring, and there are 13 systems that the Sn function gets higher RMSD than the RP scoring.

**Figure 10:**
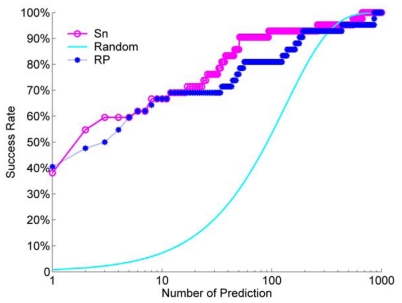
For the bound dock results, the comparison of the success rate between the Sn scoring function, RP scoring function and a random rank.

For bounded docking decoy, we also compared the success rate between the Sn scoring function, RP scoring function and a random rank. The result is shown in Figure 10.

From Figure 10, we can find that, for a scoring rank of bound decoys, the Sn has a better performance than the RP scoring function for most of NP. Moreover, the Sn and RP scoring functions all get a better result than the random rank for a small NP. Due to the monomer structure from the complex, the docking will get more near native structures, so the success rate of a bound docking is obviously higher than that of an unbound docking. When we select the top 10 decoys from the scoring rank queue, or the NP is equal to 10, the success rate is about 60%. It is about two times that of the unbound docking.

Because the hit number of a bound docking is bigger than that of an unbound docking, the random rank of bound decoys set will get a better performance than a random rank for a set of unbound docking decoys.

From the comparison of the results mentioned above, we can conclude that Sn also has a similar discriminative ability with the RP scoring function on the scoring and ranking of the bounded docking decoy.

On the different group, the same scoring function has a different performance. The results of Sn on the Enzyme-Inhibitor are better than that of the ‘others’ type. The highest correlation coefficients between the scoring values and L_RMSD is 0.7, obtained from the 1udi system in the Enzyme-Inhibitor group. The main reason for this phenomenon is that the ‘others’ complex is a kind of structure which is difficult to research in the docking. The ‘others’ complex often holds an important role in the signal transduction or in the synergistic effect of organism. They have the essential characteristic of drug identification targets. This type of complex has a great theoretical research value and a potential application prospect. However, if the conformation change before and after the binding is great, then the sampling and the scoring all have certain difficulties in the docking process.

## 5. Conclusions

Based on the weighted residue network, we proposed a new docking scoring function Sn. With this scoring function, we do the scoring rank for 42 systems’ bound and unbound docking results. Comparing with the results obtained from the RP scoring function, we find that the Sn scoring function has a similar performance with the RP on four items. On some special systems, or on some indicators, this new scorning function has a better performance. When comparing the success rate, Sn has a better performance than RP. So, we can conclude that Sn has a higher power to pick the hit out than RP.

Compared with other types scoring function, the advantage of this new scoring function is the simplicity and clearness of its calculation. It does not need a heavy computation, but the scoring rank result is acceptable.

Furthermore, with the weighted residue network model, the global topological characteristics of the protein-protein complex can be considered in this scoring function. The detail of the interaction between residues, containing the interaction modes and interaction strength, will be taken into account in the calculation of this scoring function. It is helpful for the explanation of the structure mechanisms for protein-protein interactions.

In this work, we only test this new scoring function with 42 single-chain monomer structures. Actually, it can be used to evaluate multi-chain protein complexes.

This scoring function can be used as a scoring item for a combinational scoring function, and the related work is undergoing. There are some new scoring methods, and some relative works have been a very big inspiration to our work [11]. In our future work, we will improve our method and do the comparison with these new pairwise propensities.
